# Effect of Process Variables on Rice Flour Functional Properties, and Porous Structure of Rice and Wheat-Based Leavened Food Products

**DOI:** 10.17113/ftb.60.01.22.7238

**Published:** 2022-03

**Authors:** Heshani Anupama Rathnayake, Senevirathne Navaratne, Champa Navaratne

**Affiliations:** 1 Department of Food Science and Technology, Faculty of Applied Sciences, University of Sri Jayewardenepura, Gangodawila, 10250, Nugegoda, Sri Lanka; 2 Department of Agricultural Engineering, Faculty of Agriculture, University of Ruhuna, Mapalana, 81100, Kamburupitiya, Sri Lanka

**Keywords:** factorial design, dry and wet grinding, heat-moisture treatment, particle size determination, porous crumb structure, rice/wheat composite flour

## Abstract

**Research background:**

Various processing techniques significantly affect physicochemical and functional properties of rice flour and the quality of the final products. This study aims to modify rice flour with different treatments and to select the best one to develop rice and wheat-based leavened food products.

**Experimental approach:**

Eight treatment combinations were applied on rice flour according to 2^3^ factorial design considering three variables at two levels, namely, pretreatment of rice grain (heat-moisture treatment, dual modification treatment: soaking of rice grains in NaHCO_3_ solution followed by heat treatment), grinding method (dry or wet grinding), and flour particle size (75−180 and <75 µm). Eight dough samples were prepared by mixing 50 g rice flour from each treatment with 50 g wheat flour. Then, the dough samples were subjected to fermentation and gelatinization under pressure (externally applied 1.0 kg/cm^2^ initial air pressure) in a pressure adjustable chamber.

**Results and conclusions:**

Rice flour sample with particle size of 75-180 µm that underwent heat-moisture treatment followed by wet grinding improved the gas retention capacity of the leavened dough. With the externally applied initial air pressure of 1.0 kg/cm^2^, we obtained highly porous and better textured rice and wheat-based leavened food products.

**Novelty and scientific contribution:**

Rice flour can be modified using the described method to improve its functional properties, and the textural and structural properties of rice and wheat-based leavened food products. Also, conducting fermentation and gelatinization under pressure is a novel food processing technique, which contributes to restricting the escape of gas from leavened rice/wheat composite dough mass.

## INTRODUCTION

Rice (*Oryza sativa* L.) is the staple food for about 50% of the world population, covering the majority in Asian countries (*1*). Rice flour is commonly used in various novel as well as traditional food products (*2*). Generally, well-developed porous crumb structure, better physicochemical properties and acceptable sensory properties are the main quality parameters that are usually considered in evaluation of the quality of leavened food products. Due to the unavailability of gluten proteins, dough samples prepared from rice flour have a poor ability to form a viscoelastic structure that entraps gas during fermentation. Hence, the development of well-porous crumb structured leavened food products from rice and other non-glutinous flour is a real challenge. Besides, rice variety, storage conditions, grinding method, flour particle size, damaged starch, protein and amylose contents, starch modification method, *etc*. can also affect the physicochemical and functional flour properties, thereby affecting the quality and the consistency of the finished products (*3*–*5*). In certain instances, starch modification techniques are also applied in the bakery industry to overcome some unfavourable properties in rice flour/starch to improve the quality of the finished products (*6*).

Heat-moisture treatment of starch is a physical modification method, where a starch and water combination is thermally treated to alter the physicochemical and functional properties of the starch, such as hydration and pasting properties, crystallinity, colour and gelatinization enthalpy (*7*–*9*). As proposed by Navaratne (*10*), when rice grains are subjected to heat-moisture treatment, water molecules penetrate into rice kernels during the period of moisture equilibration. This causes water molecules to bind with more glucose units (available in starch) by hydrogen bonds through the vibration energy caused during rapid heat treatment (80–85 °C for 5 min). Under these conditions, rice starch is gelatinized and becomes stickier. Hence, flour subjected to heat-moisture treatment is more suitable for making bakery products. Additionally, Arns *et al.* (*11*) also observed that heat-moisture treated rice grains required lower gelatinization temperature and higher pasting temperature.

A combination of both physical and chemical starch modifications (dual modification) can also be applied to improve the physicochemical and functional properties of flour. Peries *et al.* (*12*) have shown that soaking rice grains in 1 M NaHCO_3_ solution for 1 h followed by washing thoroughly and keeping in an electric oven at 100 °C for 2 h improves functional properties of rice flour. Furthermore, they mixed 40% rice flour with 60% wheat flour to develop bread with more acceptable organoleptic properties. Generally, NaHCO_3_ added to soaking water can soften the rice kernels and absorb into rice grains. The absorbed NaHCO_3_ causes the release of CO_2_ during the heat treatment. The release of CO_2_ facilitates the cracking of the rice grains. Cracks formed in rice grains enable the formation of fine particles during grinding. Dual modification treatment requires a longer heat treatment than heat-moisture treatment to facilitate the formation of cracks on rice grains and to reduce their moisture content (because the initial moisture content of the rice grains after dual modification is high due to immersion in NaHCO_3_ solution). However, previous studies have not disclosed how the rice grains subjected to heat-moisture treatment or dual modification treatment combined with grinding method and particle size would affect the fuctional flour properties and the formation of porous crumb structure of rice and wheat-based leavened food products.

According to our previous study (*13*), fermentation and gelatinization under an externally applied 1.0 kg/cm^2^ initial air pressure in a hermetically sealed container contribute to the increase of gas retention capacity of rice and wheat-based leavened dough samples (rice/wheat 50:50). Besides, the obtained product showed a stable and less brittle crumb structure with a uniform crumb cell distribution.

The objective of this study is to modify rice flour using different treatments such as pretreatments of rice grains (heat-moisture treatment and dual modification treatment), size reduction by adapting two grinding methods (wet and dry grinding), and using two sizes of rice flour particles (75-180 and <75 µm). Furthermore, we evaluated flour gel hydration properties and improved porous structure properties of rice and wheat-based leavened food products prepared under 1.0 kg/cm^2^ (externally applied) initial air pressure.

## MATERIALS AND METHODS

### Materials

Certified paddy (BG 300) was obtained from the Rice Research and Development Institute, Bathalagoda, Sri Lanka. Refined wheat flour was purchased from a local supermarket in Colombo, Sri Lanka (Prima Ceylon; moisture 13.20%, protein (dry basis) 11.10%, particle size <180 µm). The refined wheat flour used in the current study was obtained from the same batch. Therefore, the effect of wheat flour on the properties of the crumb samples was assumed as constant. Other major ingredients used in this study include baker’s yeast (Mauripan Instant Dry Yeast, AB Mauripan, Balakong, Malaysia), table salt (Raigam Eastern Salt Co. Ltd, Puttalam, Sri Lanka), sugar, shortening (Pyramid Wilmar Pvt Ltd, Colombo, Sri Lanka) and NaHCO_3_ (E500(ii); AB Mauri, Balakong, Malaysia).

Polyethylene terephthalate/linear low density polyethylene (PET/LLDPE) polymer bags (10 micron P/PET, 50 micron P/LLDPE) were purchased from Acme Printing and Packaging PLC, Piliyandala, Sri Lanka.

### Preparation of rice flour samples according to 2^3^ factorial design

Paddy was dehusked and polished thoroughly. Then, eight batches of rice flour were prepared according to 2^3^ factorial design considering three variables at two levels, namely pretreatment of rice grains (heat-moisture treatment (HMT) and dual modification treatment), grinding technique (dry and wet grinding) and flour particle size (75−180 µm and <75 µm) as shown in Table 1. The particle size fractions were selected based on previous studies (*14*, *15*).

**Table 1:** Eight rice flour treatment combinations according to 2^3^ factorial design

**Treatment number**	**Treatment combination**		
Pretreatment	Grinding	*d*(particle)/µm
**1**	HMT	dry	75−180
**2**	HMT	dry	<75
**3**	HMT	wet	75−180
**4**	HMT	wet	<75
**5**	DMT	dry	75−180
**6**	DMT	dry	<75
**7**	DMT	wet	75−180
**8**	DMT	wet	<75

HMT=heat-moisture treatment, DMT=dual modification treatment

#### Heat-moisture treatment of rice grains

Rice grains were subjected to heat-moisture treatment according to the method described by Navaratne (*10*) with slight modifications. A calculated amount of water was added to rice grains (g) according to the following equation:


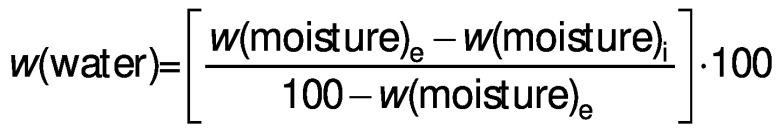



where *w*(moisture)_e_ is equilibrium moisture content that needs to be obtained, *w*(moisture)_i_ is initial moisture content of rice grains and *w*(water) is the amount of water (g) that needs to be added to 100 g of rice.

The rice and water were mixed well to make the moisture content of rice around 16.5-17.0%. Subsequently, rice was packed in an airtight, clean, double-laminated PET/LLDPE bag and kept under ambient conditions ((29±2) °C, 68% relative humidity (RH)) for 18-24 h to get the rice at equilibrium moisture content. Then, it was treated at (85±2) °C for 4-5 min in a laboratory-scale rotary dryer (Advanced Engineers International Pvt Ltd, Boralesgamuwa, Sri Lanka).

#### Dual modification treatment for rice grains

Rice grains were subjected to chemical and physical modification according to the method described by Peries *et al.* (*12*) with minor alteration. Dehusked and well-polished rice grains were dipped in 1 M NaHCO_3_ solution for 1 h, then washed thoroughly and thermally treated in the laboratory-scale rotary dryer (Advanced Engineers International) at (84±2) °C for 1 h.

#### Dry grinding and sieving

A portion of the treated rice grains was ground (MX-110PN; National, Osaka, Japan) and sieved through 180 and 75 µm sieves using a sieve shaker (Minor 200; Endecotts, London, UK) to obtain rice flour with particle sizes between 75-180 µm or smaller than 75 µm.

#### Wet grinding and sieving

A portion of the treated rice grains was soaked in clean water ((25±2) °C) for 4 h, the excess water was drained and the grains were ground (MX-110PN; National). Rice flour was dried (universal oven UN30; Memmert GmbH, Büchenbach, Germany) at (55±1) °C for 8 h, and passed through 180 and 75 µm sieves.

### Preparation of crumb samples

Eight crumb samples were prepared from each treatment (Table 1) according to the method of Rathnayake *et al*. (*13*). Rice and wheat composite flour (rice flour/wheat flour 50:50), salt (1%) and yeast slurry (which contained dry yeast 2%, sugar 1.6%) were mixed while adding lukewarm ((40±2) °C) water. The dough was kneaded (SB-08L planetary mixer; Sherry, Taichung, Taiwan) for 6 min and then shortening (2%) was added while continuing the kneading for another 1 min. Small dough mass of (35.00±0.05) g (6 dough portions per each treatment) were weighed and placed in cylindrical sample containers. A thin shortening layer was applied over the dough surface to prevent the case hardening due to the ambient temperature and RH. Next, the dough samples were fermented inside a hermetically sealed chamber under externally applied air pressure at 1.0 kg/cm^2^. After fermentation for 180 min, the chamber with the samples was heated for 15 min while releasing the pressure inside the chamber gradually, parallel to the starch gelatinization process.

### Analysis of flour gel hydration properties

A mass of (0.500±0.002) g of each flour type was dispersed in 10 mL distilled water and agitated in a shaking water bath (BIS-2 water bath incubator shaker; Microsil, Ambala Cantonment, India) at (80±2) °C for 30 min. Then, the cooked paste was cooled in an ice bath for 10 min and centrifuged (Z 306 universal centrifuge; HERMLE Labortechnik GmbH, Wehingen, Germany) at 3000 rpm for 10 min. The dry solid mass was obtained by evaporating the supernatant overnight at (105±2) °C (UN30 oven; Memmert GmbH). Water absorption index (WAI), water solubility index (WSI) and swelling power (SP) were calculated using the respective equations below (*3*):


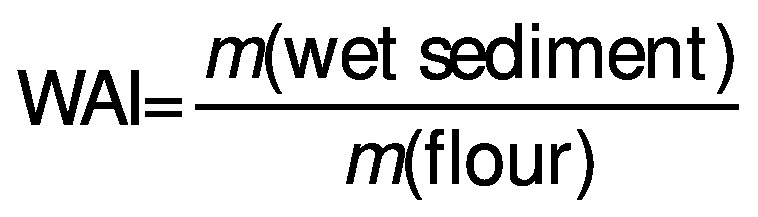




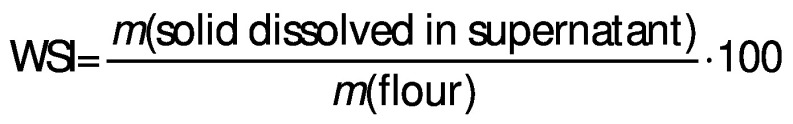




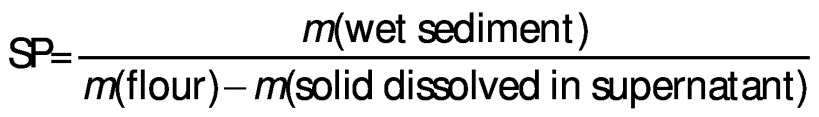



### Light microscopy examination

For light microscopy examinations, the flour samples from each treatment were suspended in aqueous glycerol (*φ*(glycerol)=0.5). A drop of each sample suspension was mounted on a slide and air-dried. The dried samples were stained with iodine and examined with a light microscope (B-292; Optika S.r.l., Ponteranica, BG, Italy) at 400× magnification. The microscopic images were acquired using a 20-megapixel camera.

### Crumb volume, specific volume and bulk density

The gelatinized crumb samples were cooled at room temperature ((30±1) °C), 68% RH) for (25±3) min and weighed. Volume of the gelatinized crumb samples was determined following the seed displacement method according to Aplevicz *et al.* (*16*). Then, specific volume (in cm^3^/g) and bulk density (in g/cm^3^) of the crumb samples were calculated.

### Crumb texture profile analysis

Crumb samples were cooled at room temperature ((30±1) °C, 68% RH) for (90±3) min and texture profile analysis was conducted using CT3 texture analyser (AMETEK Brookfield, Middleboro, MA, USA) along with TexturePro CT software (*17*), according to Wang *et al.* (*18*) and Angioloni and Collar (*19*) with some modifications including (20±1) mm sample height, 25 mm probe diameter (model TA11/1000), two compression cycles, penetration depth 50%, trigger load 5 g, test speed 1 mm/s and cell load 4500 g.

### Crumb cellular structure (image analysis)

Crumb ((3±0.5) mm thickness) cellular structure, namely crumb porosity (%), cell density (cell/cm^2^), average cell area (cm^2^), cell circularity, and solidity of the scanned images (300 dpi; flatbed scanner CanoScan LiDE-120; Canon, Tokyo, Japan) were evaluated using ImageJ v. 1.51j8 software (*20*). The scanned images (30 scanned images from each type of the sample) were converted into 8-bit (grayscale), manually thresholded (according to the histogram of grey-level frequencies), and crumb cellular structure was compared to a pre-set scale (cm).

Crumb morphological structure (fractal dimension) of the 8-bit threshold images was analysed following the box-counting method using ImageJ v. 1.51j8 software (*20*) according to Pérez-Nieto *et al*. (*21*).

### Statistical analysis

The data obtained in the study were analysed using Minitab v. 17.1.0 statistical software (*22*). All the trials were conducted in triplicate and the mean and standard deviation were calculated for each treatment. Analysis of variance (factorial ANOVA) followed by Tukey’s pairwise sample comparison were used to compare the mean values at a 95% confidence level. All the graphs were drawn using Microsoft Excel 2013 (*23*).

## RESULTS AND DISCUSSION

### Flour gel hydration properties

**Fig. 1:**
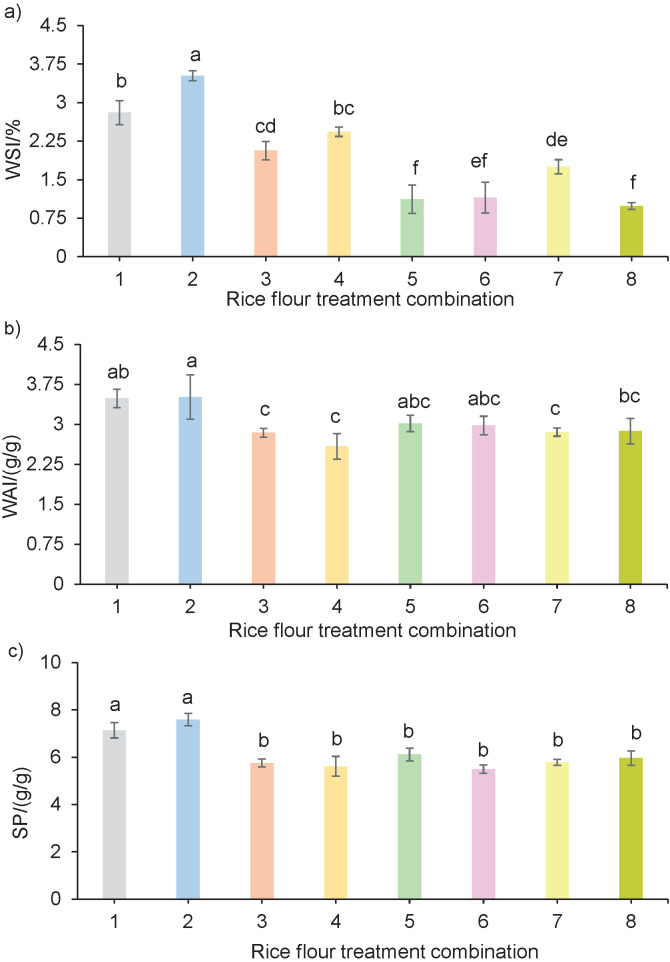
Gel hydration properties of the rice flour obtained in eight treatments: a) water solubility index (WSI), b) water absorption index (WAI) and c) swelling power (SP). Results are represented as mean value±S.D. (*N*=3). Mean values in the same plot with different letters in superscript are significantly different at α=0.05 significance level. Rice flour treatment combinations: (1) HMT, dry grinding, *d*(particle)=75−180 µm, (2) HMT, dry grinding, *d*(particle)<75 µm, (3) HMT, wet grinding, *d*(particle)=75−180 µm, (4) HMT, wet grinding, *d*(particle)<75 µm, (5) DMT, dry grinding, *d*(particle)=75−180 µm, (6) DMT, dry grinding, *d*(particle)<75 µm, (7) DMT, wet grinding, *d*(particle)=75−180 µm, and (8) DMT, wet grinding, *d*(particle)<75 µm. HMT=heat-moisture treatment, DMT=dual modification treatment

Results of the analysis of flour gel hydration properties (water absorption index (WAI), water solubility index (WSI) and swelling power (SP)) in the eight treatments are given in Fig. 1. Comparison of WSI (Fig. 1a) and SP (Fig. 1c) of the eight rice flour samples indicated a statistically significant interaction (p<0.05) between the three factors (*i.e.* pretreatment, grinding method and particle size). In addition, pretreatment and grinding showed a significant influence (p<0.05) on WAI values of the eight rice flour treatments (Fig.1b). However, when comparing the overall results of functional properties (Fig. 1), rice flour with particle size <75 µm obtained after heat-moisture treatment and dry grinding had the highest WAI, WSI and SP values.

**Fig. 2:**
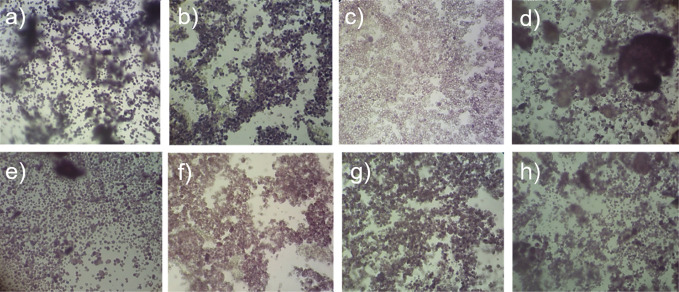
Light microscopy examinations of rice flour obtained after eight treatments (magnification: 400×): a) HMT, dry grinding, *d*(particle)=75−180 µm, b) HMT, dry grinding, *d*(particle)<75 µm, c) HMT, wet grinding, *d*(particle)=75−180 µm, d) HMT, wet grinding, *d*(particle)<75 µm, e) DMT, dry grinding, *d*(particle)=75−180 µm, f) DMT, dry grinding, *d*(particle)<75 µm, g) DMT, wet grinding, *d*(particle)=75−180 µm, and h) DMT, wet grinding, *d*(particle)<75 µm. HMT=heat-moisture treatment, DMT=dual modification treatment

Fig. 2 shows the light microscopy examinations of the eight rice flour treatments (magnification: 400×).

Leewatchararongjaroen and Anuntagool (*4*), Heo *et al*. (*24*) and Yoenyongbuddhagal and Noomhorm (*25*) stated that the values obtained for water absorption, water solubility and swelling power of wet-ground rice flour were lower than of dry-ground rice flour at temperatures below gelatinization and higher at temperatures above gelatinization. According to the results of this study, a significantly high interaction (p<0.05) of all parameters (WSI, WAI and SP) was observed among dry-ground samples compared to wet-ground samples. The *post hoc* tests with Tukey’s comparisons showed that dry-ground flour samples with particle size <75 µm had significantly higher (p<0.05) WSI values than wet-ground flour samples with particle size 75−180 µm. Generally, during dry grinding, a comparatively considerable amount of starch granules are damaged due to high mechanical stress and heat energy (*24*, *26*). When grinding to obtain finer flour particles, higher energy consumption causes greater damage to the starch granules (*27*). Therefore, rice flour with finer particles had higher water absorption, water solubility and swelling power than the rice flour with coarser particles (*24*, *28*). In the case of WSI, rice flour subjected to heat-moisture treatment showed a statistically significant interaction (p<0.05) with both grinding method and particle size compared to dual modification treatment. Thus, this indicates that the dual modification treatment significantly (p<0.05) reduced the WSI of the rice flour samples compared to the rice flour samples subjected to heat-moisture treatment.

Swelling power of flour can highly depend on the water-holding capacity of the starch through hydrogen bonds, because hydrogen bonds between starch molecules can be broken with the starch gelatinization and starch molecules can be replaced by water molecules (*4*, *29*). Fig. 1c shows that rice flour with particle size 75−180 or <75 µm obtained by heat-moisture treatment and dry grinding had the highest swelling power among all the eight treatment combinations, indicating a statistically significant (p<0.05) interaction.

### Physical properties of the crumb samples

Physical properties of the eight rice and wheat-based crumb samples (gelatinized) from eight rice flour treatments were compared in order to determine the best treatment parameters based on the gas retention capacity, and textural and structural properties of the crumb samples.

The values of the volume, specific volume and bulk density of eight crumb samples in Table 2 show that all three factors (*i.e.* pretreatment, grinding method and particle size) have a statistically significant interaction (p<0.05). According to *post hoc* tests with Tukey’s comparisons, crumb sample prepared from rice flour with particle size 75−180 µm subjected to heat-moisture treatment and then wet ground had the highest crumb volume, specific volume and the lowest bulk density compared to the other crumb samples (all p<0.05). These characteristics demonstrate that the combination of heat-moisture treatment with wet grinding, and particle size 75−180 µm result in the best gas retention capacity of leavened dough.

**Table 2:** Volume, specific volume and bulk density of the crumb samples prepared in eight different rice flour treatments

**Rice flour treatment**			*V*(crumb)/cm^3^	*v*(crumb)/(cm^3^/g)	*ρ*_b_(crumb)/(g/cm^3^)
**Pretreatment**	Grinding		**	**	**
**HMT**	dry		45.101^c^	1.640^c^	0.625^a^
**HMT**	wet		54.564^a^	1.951^a^	0.518^c^
**DMT**	dry		44.750^c^	1.580^d^	0.633^a^
**DMT**	wet		48.640^b^	1.734^b^	0.580^b^
**Pretreatment**	*d*(particle)/µm		**	**	**
**HMT**	75−180		54.723^a^	1.966^a^	0.510^c^
**HMT**	<75		44.943^c^	1.625^c^	0.633^a^
**DMT**	75−180		48.343^b^	1.733^b^	0.581^b^
**DMT**	<75		45.047^c^	1.581^d^	0.633^a^
**Grinding**	*d*(particle)/µm		--	--	--
**dry**	75−180		48.052^b^	1.723^b^	0.584^b^
**dry**	<75		41.800^c^	1.498^c^	0.674^a^
**wet**	75−180		55.013^a^	1.976^a^	0.507^c^
**wet**	<75		48.191^b^	1.708^b^	0.591^b^
**Pretreatment**	Grinding	*d*(particle)/µm	**	**	**
**HMT**	dry	75−180	(51.2±0.4)^b^	(1.85±0.02)^b^	(0.541±0.006)^c^
**HMT**	dry	<75	(39.0±0.8)^d^	(1.43±0.02)^d^	(0.71±0.02)^a^
**HMT**	wet	75−180	(58.2±0.8)^a^	(2.08±0.03)^a^	(0.480±0.007)^d^
**HMT**	wet	<75	(50.9±0.8)^b^	(1.82±0.01)^b^	(0.56±0.01)^c^
**DMT**	dry	75−180	(44.9±0.6)^c^	(1.6±0.02)^c^	(0.627±0.006)^b^
**DMT**	dry	<75	(44.6±0.4)^c^	(1.6±0.01)^c^	(0.64±0.01)^b^
**DMT**	wet	75−180	(51.8±0.3)^b^	(1.87±0.01)^b^	(0.534±0.004)^c^
**DMT**	wet	<75	(45.4±0.8)^c^	(1.6±0.05)^c^	(0.63±0.02)^b^

Mean values in the same column with different letters in superscript are significantly different at α=0.05 according to factorial ANOVA followed by Tukey’s pairwise comparison. **Interaction effect is significant at α=0.05, --interaction effect is not significant at α=0.05 significance level. HMT=heat-moisture treatment, DMT=dual modification treatment

Furthermore, results in Table 2 demonstrate that the crumb samples of the rice flour subjected to wet grinding had higher crumb volume, specific volume and lower bulk density values than the rice flour obtained by dry grinding with the same pretreatment due to the significant interaction (p<0.05) between the pretreatment and grinding method. Also, there was a significant interaction (p<0.05) between the particle size and the pretreatment. This indicates that crumb samples of the rice flour with finer particles (<75 µm) had lower crumb volume, specific volume and higher bulk density values than the crumb samples of the rice flour with the same pretreatment and the particle sizes of 75−180 µm. De la Hera *et al.* (*15*) also observed that bread prepared from short grain rice flour with particle size between 106 and 180 µm had comparatively higher crumb specific volume than the other samples with particle sizes >180, 106−80 and <80 µm.

Texture profile of a leavened food product mainly depends on the development of crumb cellular structure. These attributes play a vital role in determining the consumer perception of the product quality. Food texture is divided into three categories, namely visual texture, auditory texture and tactile texture. Among them, the tactile texture is the most commonly considered textural property of a food product and is defined as the sensation of direct contact between the food and human skin either by hand or by oral surface (*30*, *31*). Table 3 shows the results of the texture profile analysis of the eight crumb samples prepared using different treatments. According to these findings, a statistically significant interaction (p<0.05) can be observed among all three factors (*i.e.* pretreatment, grinding method and particle size) in crumb hardness, gumminess and chewiness. Therein, the crumb sample prepared with the flour of particle size 75−180 µm subjected to heat-moisture treatment and then wet ground showed the lowest values compared to the other crumb samples.

**Table 3:** Texture profile analysis of the crumb samples prepared from eight rice flour treatment combination

**Rice flour treatment**			Hardness/N	Springiness/mm	Cohesiveness	Gumminess/N	Chewiness/mJ
**Pretreatment**	Grinding		**	--	**	**	**	
**HMT**	dry		34.13^a^	9.755^a^	0.485^b^	16.31^a^	163.831^a^	
**HMT**	wet		15.13^d^	9.849^a^	0.548^a^	8.31^c^	82.018^c^	
**DMT**	dry		26.00^c^	9.755^a^	0.563^a^	13.82^b^	134.873^b^	
**DMT**	wet		28.41^b^	9.622^a^	0.571^a^	15.51^a^	146.517^b^	
**Pretreatment**	*d*(particle)/µm		**	**	**	--	**	
**HMT**	75−180		16.33^c^	9.729^ab^	0.545^b^	8.87^d^	85.912^c^	
**HMT**	<75		32.93^a^	9.8751^a^	0.488^c^	15.76^b^	159.938^a^	
**DMT**	75−180		22.17^b^	10.020^a^	0.558^ab^	11.79^c^	119.788^b^	
**DMT**	<75		32.24^a^	9.413^b^	0.576^a^	17.54^a^	161.602^a^	
**Grinding**	*d*(particle)/µm		**	--	**	**	**	
**dry**	75−180		20.93^c^	9.924^a^	0.548^a^	11.02^c^	110.871^c^	
**dry**	<75		39.19^a^	9.642^a^	0.500^b^	19.12^a^	187.834^a^	
**wet**	75−180		17.56^d^	9.825^a^	0.555^a^	9.64^d^	94.828^c^	
**wet**	<75		25.97^b^	9.645^a^	0.564^a^	14.18^b^	133.707^b^	
**Pretreatment**	Grinding	*d*(particle)/µm	**	--	**	**	**	
**HMT**	dry	75−180	(19.87±1.88)^de^	(9.7±0.4)^ab^	(0.52±0.03)^c^	(10.48±1.45)^de^	(101.2±10.7)^cd^	
**HMT**	dry	<75	(48.39±1.54)^a^	(9.8±0.3)^ab^	(0.45±0.01)^d^	(22.15±0.32)^a^	(226.5±8.8)^a^	
**HMT**	wet	75−180	(12.79±0.92)^f^	(9.8±0.6)^ab^	(0.57±0.03)^ab^	(7.26±0.34)^f^	(70.6±2.9)^e^	
**HMT**	wet	<75	(17.47±0.54)^e^	(9.9±0.2)^ab^	(0.52±0.04)^c^	(9.37±0.78)^e^	(93.4±7.9)^de^	
**DMT**	dry	75−180	(22.00±2.01)^d^	(10.2±0.4)^a^	(0.58±0.02)^ab^	(11.56±0.73)^d^	(120.6±15.2)^c^	
**DMT**	dry	<75	(29.99±1.91)^c^	(9.5±0.3)^b^	(0.550±0.008)^bc^	(16.09±1.46)^c^	(149.2±16.4)^b^	
**DMT**	wet	75−180	(22.33±2.45)^d^	(9.9±0.3)^ab^	(0.54±0.006)^bc^	(12.02±1.38)^d^	(119.0±15.3)^c^	
**DMT**	wet	<75	(34.48±1.96)^b^	(9.4±0.4)^b^	(0.60±0.01)^a^	(19.00±2.12)^b^	(174.0±26.4)^b^	

Mean values in the same column with different letters in superscript are significantly different at α=0.05 according to factorial ANOVA followed by Tukey’s pairwise comparison. **Interaction effect is significant at α=0.05 significance level, --interaction effect is not significant at α=0.05 significant level. HMT=heat-moisture treatment, DMT=dual modification treatment

Springiness is the ability of a sample to bounce back to its original position after completing a compressive force (*32*, *33*). Eight crumb samples did not show a significant interaction (p>0.05) among the three factors (Table 3). However, crumb samples prepared from rice flour treated with dual modification with particle size 75−180 µm showed the highest values of springiness.

Results further indicated that the crumb sample prepared using dual modification treatment and wet grinding, with particle size <75 µm had the highest cohesiveness value, as shown in Table 3. However, this value is not significantly different (p>0.05) from the cohesiveness value of the crumb sample with particle size 75−180 µm prepared by heat-moisture treatment and wet grinding. This high cohesiveness value also proves that rice flour obtained by this treatment imparts higher specific volume and lower crumb hardness.

According to Araki *et al.* (*34*), if the amount of damaged starch granules is low, the WAI of rice flour is also low and the bakery products prepared from these types of flour can result in higher crumb specific volume with better crumb textural properties. The results of this study (Fig. 1, Table 2 and Table 3) also indicate that rice flour subjected to heat-moisture treatment followed by wet grinding had the lowest WAI along with the highest crumb volume and specific volume, and the lowest bulk density, hardness, gumminess and chewiness. Moreover, the results obtained in the current study further demonstrate that the combination of wet grinding and flour particle size 75−180 µm contribute to the highest (p<0.05) crumb specific volume and the lowest hardness, gumminess and chewiness in rice and wheat-based leavened products.

**Fig. 3:**
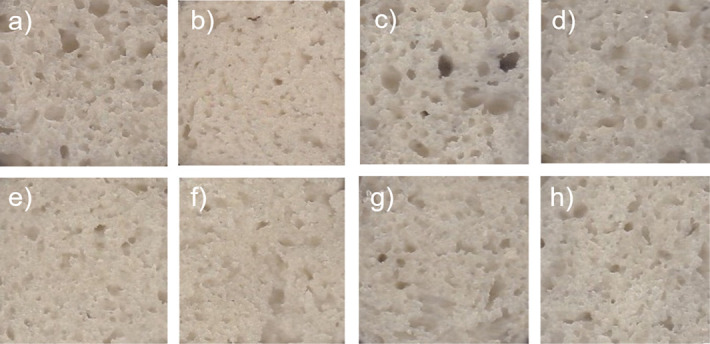
Scanned images (300 dpi) of the porous structure of the rice flour crumb obtained after eight treatments: a) HMT, dry grinding, *d*(particle)=75−180 µm, b) HMT, dry grinding, *d*(particle) 75 µm, c) HMT, wet grinding, *d*(particle)=75−180 µm, d) HMT, wet grinding, *d*(particle)<75 µm, e) DMT, dry grinding, *d*(particle)=75−180 µm, f) DMT, dry grinding, *d*(particle)<75 µm, g) DMT, wet grinding, *d*(particle)=75−180 µm, and h) DMT, wet grinding, *d*(particle)<75 µm. HMT=heat-moisture treatment, DMT=dual modification treatment

When a leavened baked product is sliced, a two-phase soft cellular solid structure can be seen. This includes a solid phase made out of the cell wall structure along with a fluid phase that consists of air (*35*, *36*). Recently, image analysis has become an important quantitative tool to reliably assess the microstructural features of crumb samples and the relationship of crumb cellular structure properties with the mechanical and organoleptic properties of the product (*35*, *37*). Fig. 3 shows scanned images (300 dpi) of the crumb samples of rice flour obtained after eight different treatments. The results of crumb cellular structure analysis (obtained by image analysis) of the eight crumb samples are shown in Table 4.

**Table 4:** Cellular structure properties (obtained by image analysis) of the crumb samples of rice flour samples from eight different treatments

**Rice flour treatment**			Porosity/%	Cell density/(cell/cm^2^)	*Ā*(cell)/cm^2^	Circularity	Solidity	Fractal dimension
**Pretreatment**	Grinding		**	--	**	--	--	--
**HMT**	dry		24.331^c^	29.602^b^	0.009^bc^	0.492^b^	0.708^b^	1.648^b^
**HMT**	wet		32.56^a^	27.208^c^	0.012^a^	0.509^a^	0.729^a^	1.664^a^
**DMT**	dry		24.635^c^	31.723^a^	0.008^c^	0.475^c^	0.690^c^	1.652^b^
**DMT**	wet		27.843^b^	29.502^b^	0.010^b^	0.481^bc^	0.704^b^	1.648^b^
**Pretreatment**	*d*(particle)/µm		**	**	**	--	--	**
**HMT**	75−180		31.938^a^	25.562^c^	0.013^a^	0.500^a^	0.728^a^	1.658^b^
**HMT**	<75		24.954^b^	31.251^a^	0.008^b^	0.501^a^	0.709^b^	1.642^c^
**DMT**	75−180		26.318^b^	31.823^a^	0.009^b^	0.482^b^	0.701^bc^	1.658^b^
**DMT**	<75		26.159^b^	29.402^b^	0.009^b^	0.473^b^	0.693^c^	1.661^ab^
**Grinding**	*d*(particle)/µm		**	--	**	--	**	**
**dry**	75−180		27.410^b^	29.612^b^	0.010^b^	0.487^ab^	0.710^a^	1.668^a^
**dry**	<75		21.557^c^	31.715^a^	0.007^c^	0.481^b^	0.688^b^	1.632^b^
**wet**	75−180		30.846^a^	27.773^c^	0.012^a^	0.496^a^	0.718^a^	1.661^a^
**wet**	<75 µm		28.557^a^	28.937^bc^	0.011^ab^	0.494^ab^	0.714^a^	1.671^a^
**Pretreatment**	Grinding	*d*(particle)/µm	--	**	--	--	**	--
**HMT**	dry	75−180	(29.1±2.3)^bc^	(25.3±2.0)^c^	(0.012±0.002)^ab^	(0.50±0.03)^abc^	(0.72±0.01)^ab^	(1.67±0.02)^ab^
**HMT**	dry	<75	(19.6±3.2)^f^	(33.9±1.8)^a^	(0.006±0.002)^d^	(0.49±0.03)^bcd^	(0.69±0.02)^cd^	(1.62±0.02)^d^
**HMT**	wet	75−180	(34.8±3.0)^a^	(25.8±2.1)^c^	(0.014±0.003)^a^	(0.5±0.04)^ab^	(0.73±0.02)^a^	(1.67±0.01)^ab^
**HMT**	wet	<75	(30.3±1.9)^b^	(28.6±2.3)^b^	(0.011±0.003)^ab^	(0.51±0.02)^a^	(0.73±0.01)^a^	(1.66±0.02)^bc^
**DMT**	dry	75−180	(25.7±2.4)^de^	(33.9±2.4)^a^	(0.008±0.002)^cd^	(0.48±0.03)^cd^	(0.70±0.02)^cd^	(1.66±0.02)^ab^
**DMT**	dry	<75	(23.5±2.4)^e^	(29.5±2.7)^b^	(0.008±0.002)^cd^	(0.47±0.03)^d^	(0.68±0.02)^d^	(1.64±0.01)^cd^
**DMT**	wet	75−180	(26.9±2.6)^cd^	(29.7±2.6)^b^	(0.009±0.002)^bc^	(0.49±0.04)^bcd^	(0.71±0.02)^bc^	(1.65±0.01)^bc^
**DMT**	wet	<75	(28.8±2.2)^bc^	(29.3±2.6)^b^	(0.010±0.002)^bc^	(0.47±0.03)^cd^	(0.70±0.02)^cd^	(1.68±0.02)^a^

Mean values in the same column with different letters in superscript are significantly different at α=0.05 according to factorial ANOVA followed by Tukey’s pairwise comparison. **Interaction effect is significant at α=0.05 significance level, --interaction effect is not significant at α=0.05 significant level. HMT=heat-moisture treatment, DMT=dual modification treatment

According to Korczyk-Szabó and Lacko-Bartošová (*36*) and Lassoued *et al.* (*38*), a well-developed porous crumb structure is represented by having a higher porosity along with a finer and regular gas cell structure. Crumb porosity is the average ratio of the total cells to the total area in a predetermined sample volume. The higher the porosity, the higher the number of gas cells that have a diameter of >1 mm and the lower the degree of cell uniformity. Cell density and cell circularity are other important factors that assess the properties of crumb cellular structure. Generally, cell density is defined as the number of cells per unit of area, where the higher the cell density, the finer the crumb cellular structure (*39*). Cell circularity is identified as a shape factor that ranges from 0 (line) to 1 (perfect circle) to represent the shape of a gas cell (*40*). However, due to the complex mechanical behaviour of the porous crumb structure, considerable structural variations can be observed even within different slices of a single sample (*41*). The morphology of those types of objects can be evaluated through the fractal dimension, where higher fractal dimension values represent rougher or complex grey-level images (*21*, *42*).

When considering the results given in Table 4, the statistical interaction between the three factors was not significantly different (p>0.05) in crumb porosity, average cell area, cell circularity and fractal dimension. However, according to the Tukey’s pairwise comparison, crumb samples of particle size 75−180 µm prepared by heat-moisture treatment and dry grinding, samples of particle size 75−180 µm prepared by heat-moisture treatment followed by wet grinding, and samples of particle size <75 µm prepared by heat-moisture treatment followed by wet grinding had higher crumb porosity, average cell area, cell circularity, solidity and fractal dimension, and lower cell density values than the other crumb samples. Furthermore, crumb samples subjected to heat-moisture treatment followed by wet grinding showed significantly high (p<0.05) porosity and average cell area compared to the other crumb samples. Scanned images (Fig. 3) of the crumb samples also confirmed the observation that highly porous crumb structure with larger, circular and solid gas cells is obtained when rice flour is obtained by heat-moisture treatment and wet grinding.

As far as overall results of this study are concerned, they prove that rice flour of particle size 75−180 µm prepared by heat-moisture treatment followed by wet grinding can be applied successfully in developing rice and wheat-based bakery products (rice/wheat 50:50), particularly using *Oryza sativa* Indica rice varieties. Also, conducting fermentation and gelatinization of rice and wheat-based dough samples under pressure is a new approach in the baking industry, as usually these two processes are performed in open air. Because this method enables entrapment of gas during fermentation as rice flour is devoid of gluten, it improves textural and structural properties of the final product.

## CONCLUSIONS

Different treatments that include pretreatment of rice grain, grinding method and rice flour particle size remarkably affect rice flour functional properties as well as the development of the porous crumb structure of rice and wheat-based leavened food products (rice/wheat 50:50). Heat-moisture treatment of rice grains followed by wet grinding imparts better crumb volume and better texture properties of rice and wheat-based leavened food products along with a highly porous crumb structure with larger, circular and solid gas cells than the dual modification treatment and dry grinding. Moreover, size reduction by wet grinding in order to get particle size 75−180 µm also positively contributed to higher crumb volume and lower bulk density than dry grinding, which gave particle sizes <75 µm in rice and wheat-based dough samples. Altogether, this study suggests that the combination of the heat-moisture treatment with wet grinding, and particle size 75−180 µm provides an appropriate rice flour which with 50% refined wheat flour results in highly porous and better textured rice and wheat-based leavened food products with higher gas retention capacity. However, to obtain these results, fermentation and gelatinization must be performed under 1.0 kg/cm^2^ externally applied initial air pressure.
